# Role of Extrachromosomal Circular DNA in Sex Reversal of *Monopterus albus*

**DOI:** 10.3390/biom16101480

**Published:** 2026-10-12

**Authors:** Junchao Fang, Guanglve Li, Wenyin Luo, Haifeng Tian, Qiaomu Hu

**Affiliations:** 1 State Key Laboratory of Mariculture Biobreeding and Sustainable Goods, Yellow Sea Fisheries Research Institute, Chinese Academy of Fishery Sciences, Qingdao 266071, China; 15119438380@163.com (J.F.); 13659644863@163.com (G.L.); ioop4916@163.com (W.L.); 2 Yangtze River Fisheries Research Institute, Chinese Academy of Fishery Sciences, Wuhan 430223, China; tianhf@yfi.ac.cn

**Keywords:** *Monopterus albus*, extrachromosomal circular DNA, hermaphroditic fish, sex reversal, sex differentiation genes

## Abstract

Extrachromosomal circular DNA (eccDNA) serves as a vital regulator of gene expression and phenotypic plasticity. The *Monopterus albus* is a protogynous hermaphroditic fish, and the functional role of eccDNA during its female-to-male sex reversal remains poorly understood. We conducted Circle-seq analysis on gonadal tissues of super-female and male individuals and integrated our findings with previously published gonadal RNA-seq data. A total of 62,574 and 34,562 eccDNAs were, on average, identified in the two groups, and 7881 down-regulated and 2738 up-regulated differentially abundant eccDNAs were screened in male gonads. Three candidate eccDNAs, eccDNA*^Dmrt1a^*, eccDNA*^Sox9a1^* and eccDNA*^ZNF239^*, were selected for subsequent analysis. Quantitative real-time PCR was applied to detect the overall transcriptional levels of their corresponding host genes, covering transcripts derived from both genomic DNA and eccDNA. Spatiotemporal expression profiling and letrozole induction experiments revealed male-biased expression of *Dmrt1a* and *Sox9a1*, while *ZNF239* was predominantly expressed in female gonads. Complete sex reversal was observed in female *M. albus* after 90-day letrozole treatment, accompanied by elevated *Dmrt1a*/*Sox9a1* and reduced *ZNF239* transcription. Fluorescence in situ hybridization localized these transcripts in gonadal germ cells and somatic cells across developmental stages. This study verifies the production of eccDNA during gonadal development. The results indicate that *Dmrt1a* and *Sox9a1* may facilitate sex reversal and suggest that *ZNF239* is likely involved in ovarian development. This work provides novel clues for deciphering the molecular mechanism underlying sex reversal from the eccDNA perspective.

## 1. Introduction

The *M. albus* is a highly esteemed freshwater aquaculture fish species in China, favored by consumers for its delicate and delicious flesh as well as its rich nutritional value. According to statistics, the annual yield of *M. albus* in China reached as high as 355,200 tons in 2023 [1], which fully demonstrates its pivotal position in the freshwater aquaculture industry. Hermaphroditic fish can be classified into three types: protogynous (female-to-male), protandrous (male-to-female), and bidirectional sex-changers (continuous sex reversal) [2]. *M. albus* is a protogynous hermaphroditic fish, undergoing a sex reversal from female to male during its life cycle. Previous studies have shown that *M. albus* are generally female within the first two years of age. Between the ages of 2 and 3 years, female individuals start to transform into intersex and eventually into males [3,4]. Notably, we found that 3–5% of older *M. albus* have not undergone sex reversal during gonad development; these individuals are known as “super-female” and share a similar morphology with male *M. albus*. These super-female *M. albus* reach a body length of over 52 cm and produce 2000–5000 eggs, far more than the 300–500 eggs produced by ordinary female individuals [5]. Therefore, if these super-female *M. albus* could be used for artificial breeding, the yield and economic benefits of artificial fry would be increased.

Aromatase inhibitor (AI) can induce the differentiation of fish gonads towards the testis direction by reducing estrogen levels. Studies have shown that treating 2-month-old *M. albus* with an AI can successfully transform their gonads from ovaries to testes [6]. This artificially induced sex reversal is of great value in the establishment of super-female *M. albus* families. During the sex reversal process of *M. albus*, although its genomic sequence remains unchanged, significant phenotypic changes occur, suggesting that epigenetics may play a crucial role in this process. In recent years, scientists have identified multiple genes related to the sex reversal of *M. albus*, such as *Sox9*, *Amh*, *Dmrt1*, *Cyp19a*, *Foxl2*, and *Nr5a* [7,8,9,10]. These studies have provided important clues for revealing the molecular mechanisms of sex reversal in *M. albus*. However, current research still mainly focuses on the chromosomal genes.

Since extrachromosomal circular DNA (eccDNA) was first discovered in wheat embryo cells and wild boar sperm cells in the 1960s [11], with the rapid development of high-throughput sequencing technology, eccDNA has been confirmed to be widely present in animal and plant cells [12,13,14]. Unlike independently existing maternally inherited circular DNA such as mitochondrial DNA (mtDNA), eccDNA mainly originates from linear DNA breaks during processes such as DNA damage repair, gene rearrangement, and DNA replication within the cell nucleus, which then circularize to form DNA fragments containing partial or complete genes, with the ability to encode functional proteins or transcribe RNAs, thereby regulating the organism [15,16]. Regarding the size of eccDNA, the currently reported sizes range from a few hundred base pairs to several million base pairs [15,17], which may be due to differences in research methods and samples. Based on features such as origin, size, and sequence, eccDNA can be classified into four types: small polydisperse circular DNA (spcDNA), telomeric circles (t-circles), microDNA, and extrachromosomal DNA (ecDNA) [18]. For the convenience of discussion, unless otherwise specified, this paper does not make a detailed distinction among these four types. Currently, research on eccDNA is mainly concentrated in fields such as human cancers, immune diseases, and neurological and cardiovascular diseases [19], while research on livestock, poultry, and aquatic species is relatively scarce. However, existing studies have shown that eccDNA can regulate animal phenotypic traits. For example, compared with carrier pigeons, the muscle tissues of meat-type King pigeons are rich in eccDNA related to muscle-development genes, giving King pigeons more muscle content [20]. Similarly, compared with ordinary grass carp, the muscle tissues of slimming grass carp are also enriched with a large amount of eccDNA related to muscle traits, making their flesh more elastic [21]. Given the unique sex reversal phenomenon of *M. albus*, it is of great significance to study whether eccDNA is also involved in the regulation of its sex reversal process. Therefore, in this study, we used high-throughput sequencing technology to measure and comparatively analyze the eccDNA of super-female and male *M. albus*. Through gene annotation corresponding to the genomic sequences of eccDNA loci and in combination with research on the relationship between the corresponding genes and sex, we finally screened, identified, and verified eccDNAs with significant differences in abundance levels. The research results aim to deeply reveal the mechanism of eccDNA in the sex reversal of *M. albus* and provide a new research direction for the role of eccDNA in the sex reversal mechanisms of hermaphroditic fish such as *M. albus*.

## 2. Materials and Methods

### 2.1. Sample Collection

All *M. albus* samples used in this experiment were sourced from Xiantao Breeding Center, Hubei Province, and were categorized into two groups: older (over 1 year old) and younger *M. albus* (approximately 2 months old). For older *M. albus*, we selected female, intersex, male, and super-female individuals and collected their gonadal tissues. These tissues were divided into two portions: one portion was rapidly frozen in liquid nitrogen and subsequently stored at −80 °C for long-term preservation, to be used for subsequent RNA and DNA extraction; the other portion was fixed in a 4% paraformaldehyde solution for subsequent tissue sectioning and morphological observation. Younger *M. albus* were used for the letrozole (aromatase inhibitor, AI)-treatment experiment. The experiment employed an industrialized recirculating aquaculture system to ensure stable and suitable water conditions for *M. albus* growth. The water quality parameters were maintained as follows: water temperature 24 ± 1 °C, dissolved oxygen > 6 mg/L, ammonia nitrogen < 1 mg/L, nitrite < 0.1 mg/L, pH ranging from 6.5 to 8.0, and a 12 h photoperiod (08:00–20:00). The younger *M. albus* were randomly divided into a treatment group (*n* = 60) and a control group (*n* = 60), and were placed in six separate breeding tanks. The treatment group was fed aquatic worms supplemented with 500 mg/kg AI daily [6], while the control group was fed ordinary aquatic worms as a baseline control.

### 2.2. Sequencing Protocol for eccDNA in M. albus Gonads

After determining the sex of *M. albus* by observing the tissue section, DNA was extracted from the gonads of male (*n* = 3) and super-female (*n* = 3) *M. albus*. These samples were then sent to GeneCreate Biological Engineering Co., Ltd. (located in Shanghai, China) for eccDNA sequencing analysis. The detailed experimental workflow for eccDNA sequencing-based analysis is shown in Figure S1. The specific sequencing protocol is as follows:

(1) eccDNA Enrichment: The previously extracted gonadal DNA from super-female and male *M. albus* was processed using the Plasmid Mini AX kit (A&A Biotechnology, Gdynia, Poland) to enrich for eccDNA.

(2) Exonuclease Treatment: Enriched eccDNA was subjected to exonuclease treatment (Plasmid-Safe ATP-dependent DNase, Epicentre, Madison, WI, USA) at 37 °C in a heating block for continuous 6-day incubation to remove linear DNA and other non-circular DNA impurities. Additional ATP and DNase were added every 24 h (30 units per day) during the reaction.

(3) Rolling Circle Amplification (RCA): Purified eccDNA was amplified using the REPLI-g Mini Kit (Qiagen, Venlo, The Netherlands, Cat. No. 150023) to increase the quantity of eccDNA for subsequent sequencing analysis. The amplification products were then purified using AMPure XP beads (Beckman, Indianapolis, IN, USA, Cat. No. A63880) to remove impurities and unamplified DNA.

(4) Ultrasonic Fragmentation and Library Construction: The RCA products were fragmented to approximately 200–300 bp using a Biorupter (Diagenode, Liège, Belgium), and the fragmented products were recovered. Library construction was then performed using the NEBNext Ultra DNA Library Prep Kit for Illumina (New England Biolabs, San Diego, CA, USA).

(5) Sequencing: Sequencing was performed using the Illumina NovaSeq 6000 platform (Illumina, San Diego, CA, USA) with a paired-end sequencing strategy, each end having a read length of 150 bp (PE150).

### 2.3. Processing and Analysis of Raw Sequencing Data

The raw sequencing data were obtained from the Illumina NovaSeq 6000 platform. After sequencing, all raw data were filtered using Trimmomatic software (version v0.32) [22] to remove adapter sequences and low-quality sequences, with parameters: ILLUMINACLIP:TruSeq3-PE.fa:2:30:10, LEADING:3, TRAILING:3, SLIDINGWINDOW:4:15, MINLEN:36. The overall quality of the raw reads was comprehensively evaluated using Q30 (indicating a base-calling accuracy of 99.9%) as the quality control standard. High-quality clean reads were aligned to the whole-genome sequence of *M. albus* (GCF_001952655.1, assembly name M_albus_1.0) in the NCBI database using the Burrows-Wheeler Aligner-MEM (BWA-MEM) software (version v0.7.17) [23] to determine the genomic positions of eccDNA. The circular DNA analysis software Circle-Map (version v1.1.4) [24] was used with parameter filtering criteria (split reads ≥ 1) for initial eccDNA detection. To reduce potential false-positive calls, only candidates supported by at least one split read were retained for downstream analysis. The detected eccDNA were annotated using bedtools software (version v2.31.0) [25], including information on their positions, sizes, and sequence characteristics. The IGV software (version v2.4.10) was used for visualization to more intuitively display the distribution and characteristics of eccDNA in the *M. albus* genome. Differential abundance analysis of eccDNA was performed using the DESeq2 R package (version v1.36.0) based on soft-clipped read counts derived from eccDNA junction sites, with thresholds of |log2FC| > 1 and *p* < 0.05. Notably, this represents an exploratory genome-wide screening strategy; raw *p*-values without FDR correction were used solely to generate candidate loci for subsequent manual curation and experimental validation and are not intended for drawing definitive genome-wide biological conclusions. Gene Ontology (GO) and Kyoto Encyclopedia of Genes and Genomes (KEGG) pathway enrichment analyses were performed for host genes associated with differentially abundant eccDNAs to reveal the potential biological processes and functions in which eccDNA may be involved. Previously published gonadal RNA-seq data from our group (NCBI SRA BioProject accession: PRJNA1292716) were adopted for integrative comparison with eccDNA profiles.

Throughout the data analysis process, all results were presented in the form of mean ± SE. Data were analyzed using SPSS 26.0 software. For comparisons between two groups, an independent-samples *t*-test was applied; for multi-group comparisons, one-way ANOVA followed by LSD post-hoc test was used. When *p* < 0.05, the difference was considered statistically significant.

### 2.4. Screening and Identification of Differentially Abundant eccDNAs Between Males and Super-Females

From the sequencing analysis results, three eccDNA loci associated with host genes that showed significantly different eccDNA abundance between males and super-females (*p* < 0.05) were selected for further validation. To obtain accurate sequence information for these eccDNAs, DNA was extracted from the gonads of female, intersex, male, and super-female *M. albus* using the phenol-chloroform method. Briefly, 0.1 g gonadal tissue was lysed with proteinase K at 55 °C for 2 h before purification. Linear DNA was effectively removed using the Exonuclease V Reaction (NEB, Ipswich, MA, USA, Cat. No. M0345) reagent; the digestion reaction was incubated at 37 °C for 30 min followed by heat-inactivation at 70 °C for 30 min to ensure the accuracy of subsequent experiments. Subsequently, RCA amplification primers (AAAAAAAAAGGCGCGCC/GGCGCGCCTTTTTTTTT) and the phi29 DNA Polymerase kit (Qiagen, Venlo, The Netherlands) were used to amplify and enrich the eccDNAs. The RCA reaction was performed at 30 °C for 4 h and terminated at 65 °C for 10 min.

Outward PCR primers were designed at the specific junction nodes of the three eccDNAs, with the primer sequences shown in Table S1. The PCR amplification reaction system was configured as follows: 10 μL 2× Es Taq Master Mix (containing dye), 1 μL forward primer (10 μM), 1 μL reverse primer (10 μM), 1 μL RCA-amplified DNA template, and the remaining volume was supplemented with ddH_2_O to a total volume of 25 μL. The amplification program was set as follows: pre-denaturation at 94 °C for 2 min; followed by 35 cycles, each cycle including denaturation at 94 °C for 30 s, annealing at 60 °C for 30 s, and extension at 72 °C for 30 s; finally, a final extension at 72 °C for 2 min. After amplification, the target sequences in the PCR products were separated by 1% agarose gel electrophoresis in 1× TAE buffer (220 V, 13 min). Subsequently, the target fragments were recovered using the QIAquick^®^ Gel Extraction Kit (Qiagen, Venlo, The Netherlands, Cat. No. 28704). The recovered fragments were cloned into the pMD18-T cloning plasmid (Takara Bio, Kusatsu, Shiga, Japan, Cat. No. 6011) and further transformed into competent cells (Trans-5α, TransGen Biotech, Beijing, China, Cat. No. CD201-01). Heat-shock transformation was performed at 42 °C for 90 s; bacteria were recovered in antibiotic-free LB medium and cultured on ampicillin-containing LB agar plates. Single colonies were picked and cultured in ampicillin-supplemented liquid LB medium for cloning and culture. Finally, PCR amplification was performed using M13 universal primers as the primers and the culture medium containing monoclonal cells as the template. After confirming the target band by agarose gel electrophoresis, some of the monoclonal PCR products were submitted to Tsingke Biotechnology Co., Ltd. (Beijing, China) for Sanger sequencing to verify eccDNA junction sequences.

### 2.5. mRNA Expression Analysis of Candidate Host Genes During Gonadal Development and Sex Reversal in M. albus

Primers for *Dmrt1a*, *Sox9a1*, and *ZNF239* were designed (Table S1) for qRT-PCR validation. These three genes correspond to the genomic loci to which the candidate differentially abundant eccDNAs were mapped. Gonadal RNA was extracted from female, intersex, male, and super-female *M. albus*, as well as from the control and experimental groups of *M. albus* that had undergone 90 days of AI treatment. After measuring the RNA concentration using a micro-volume spectrophotometer (NanoPhotometer NP80, Implen, Munich, Germany), the extracted total RNA was reverse-transcribed into cDNA according to the instructions of the Hifair^®^ III 1st Strand cDNA Synthesis SuperMix for qPCR (with gDNA digester Plus) kit (Yeasen, Shanghai, China), for subsequent qRT-PCR validation.

The qRT-PCR reaction system was configured as follows: 10 µL of Hieff^®^ qPCR SYBR Green Master Mix, 0.4 µL of forward primer, 0.4 µL of reverse primer, 1 µL of cDNA template, with the remaining volume made up to 20 µL with ddH_2_O. The amplification program was set as follows: pre-denaturation at 95 °C for 5 min; followed by 40 cycles, each including denaturation at 95 °C for 30 s and annealing at 60 °C for 30 s; finally, a melting curve analysis was performed, including denaturation at 95 °C for 15 s, annealing at 60 °C for 1 min, and denaturation at 95 °C for 1 s. Relative gene expression was calculated using the 2^−ΔΔCt^ method.

### 2.6. Cellular Localization of Candidate Host-Gene Transcripts During Gonadal Development and Sex Reversal in M. albus

We synthesized FISH probes for *Dmrt1a*, *Sox9a1*, and *ZNF239*, with the probe sequences shown in Table S1. These target genes represent the genomic regions to which candidate eccDNAs were mapped. These probes were synthesized by Servicebio (located in Wuhan, China). The FISH procedure is briefly outlined as follows: First, paraffin-embedded gonadal tissue sections from female, intersex, male, super-female, control, and AI-treated *M. albus* were dewaxed and rehydrated. Subsequently, the sections were digested with proteinase K. After the pre-hybridization step, a hybridization solution containing fluorescently labeled target probes was added, and the sections were incubated overnight in a 40 °C constant-temperature incubator. The next day, the nuclei were counterstained with 4,6-diamidino-2-phenylindole (DAPI). Finally, a mounting medium was added, and a coverslip was placed. Cell images were captured and recorded using an OLYMPUS BX53 fluorescence microscope (Olympus, Tokyo, Japan).

## 3. Results

### 3.1. Identification of eccDNA and Differentially Abundant eccDNAs

EccDNA profiles were generated using Circle-seq. Raw reads were filtered using Trimmomatic software. To ensure the sequencing quality of eccDNA, filtered reads with a Q30 > 80% were used as the criterion for assessing base quality.

**Figure 1:**
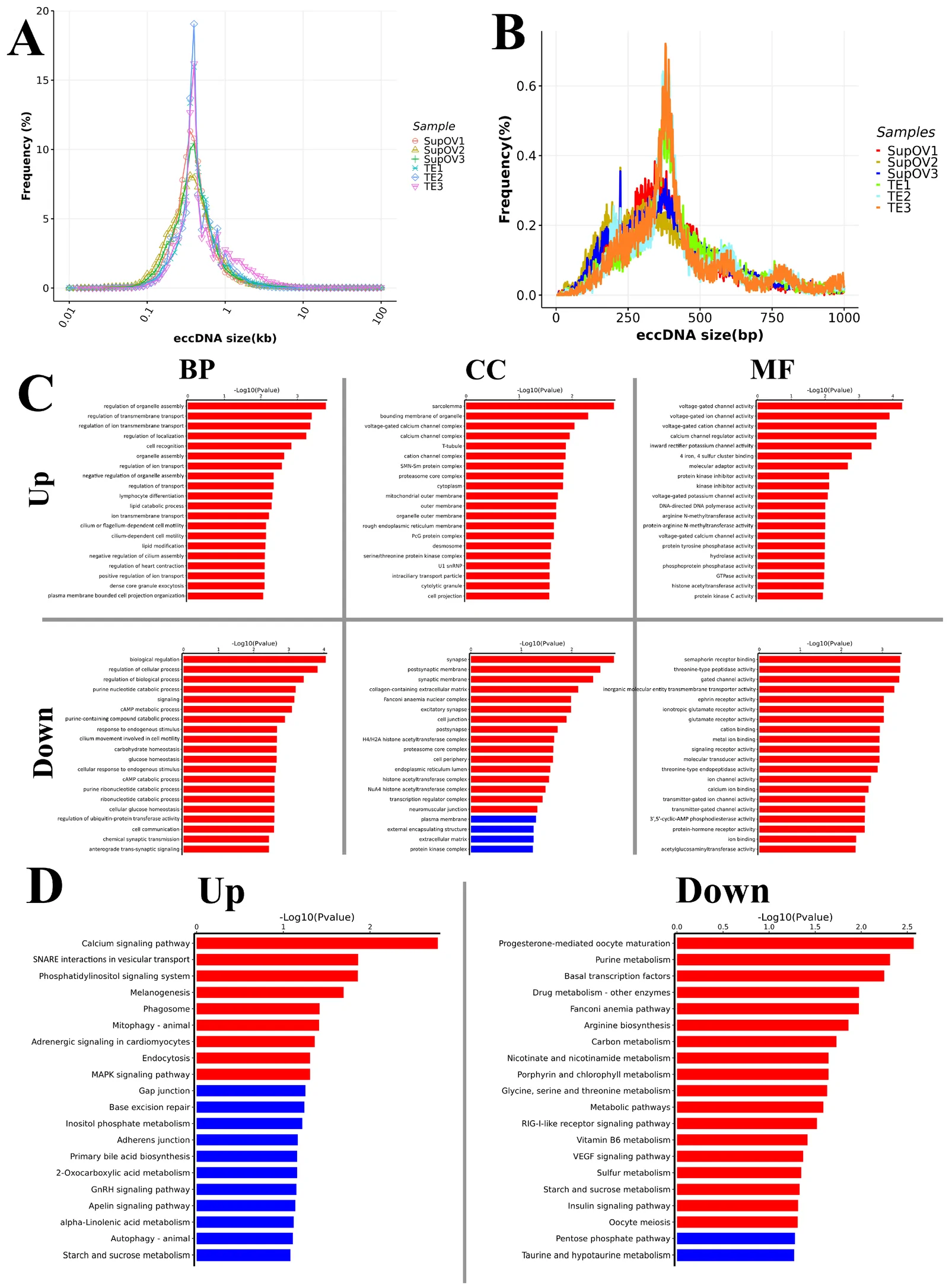
The distribution of eccDNA and the enrichment of GO and KEGG pathways in super-female and male *M. albus.* (**A**): Scatter plot of eccDNA size distribution; (**B**): Curve graph of eccDNA size; (**C**): GO enrichment analysis of genes associated with differentially abundant eccDNAs; (**D**): KEGG pathway enrichment analysis of genes associated with differentially abundant eccDNAs. BP: Biological Process; CC: Cellular Component; MF: Molecular Function; Red bars indicate significant enrichment (*p* < 0.05), while blue bars indicate insignificant enrichment (*p* > 0.05). The same colour scheme applies to subsequent figures.

A total of 138,371,756, 139,095,932, 134,531,860, 138,643,378, 144,042,580, 148,533,720 raw reads were generated from three super-female gonadal samples (SupOV1, SupOV2, SupOV3) and three male testis samples (TE1, TE2, TE3), respectively (Table 1). SupOV denotes super-female ovary; TE denotes testis. After alignment with BWA-MEM software and filtering of low-quality data using Circle-Map software, the clean data of each sample are shown in Table 1. EccDNA sizes mainly ranged from 0.1 to 1 kb, with the peak between 250 to 500 bp (Figure 1).

The detected and identified eccDNAs were subjected to gene annotation using scripts developed by the testing company and bedtools software to understand the corresponding genes of the eccDNAs. After all detected eccDNAs were annotated, the number of eccDNA-associated genes in SupOV was 20,729, 21,177, and 22,137, respectively, while for TE, it was 22,220, 22,073, and 22,291, respectively. These figures represent the total host genes overlapped by all detected eccDNAs in each sample, not differentially expressed genes. For descriptive characterization, GO and KEGG enrichment analyses were performed on the genes associated with all detected eccDNAs (Figures S2 and S3). To perform differential abundance analysis, the eccDNA loci detected in all samples were combined into a unified union set. We used bedtools software to calculate the sum of soft-clipped read counts at each eccDNA junction site across all individual samples. Further, DESeq2 was employed to calculate the differential abundance results of eccDNAs between groups, and eccDNAs with abundance level differences were screened based on the set thresholds (|log2FC| > 1, *p* < 0.05). The results indicated that, compared to super-female *M. albus*, there were 7881 down-regulated and 2738 up-regulated differentially abundant eccDNAs in male *M. albus*.

### 3.2. Functional Enrichment Analysis of Host Genes Associated with Differentially Abundant eccDNAs

We used clusterProfiler software (version v4.4.4) to calculate the significance level of gene enrichment in each GO category and performed GO enrichment analysis on host genes associated with differentially abundant eccDNAs between groups. Based on the gene functions regulated by transcription factors, GO was divided into three types: biological process (BP), cellular component (CC), and molecular function (MF).

The up-regulated and down-regulated host genes associated with differentially abundant eccDNAs were enriched in GO terms. For up-regulated GO terms, the top three significantly up-regulated BP enrichment terms were regulation of organelle assembly (GO:1902115), regulation of transmembrane transport (GO:0034762), and regulation of ion transmembrane transport (GO:0034765); the top three significantly up-regulated CC enrichment terms were sarcolemma (GO:0042383), bounding membrane of organelle (GO:0098588), and voltage-gated calcium channel complex (GO:0005891); the top three significantly up-regulated MF enrichment terms were voltage-gated channel activity (GO:0022832), voltage-gated ion channel activity (GO:0005244), and voltage-gated cation channel activity (GO:0022843). For down-regulated GO terms, the top three significantly down-regulated BP enrichment terms were biological regulation (GO:0065007), regulation of cellular process (GO:0050794), and regulation of biological process (GO:0050789); the top three significantly down-regulated CC enrichment terms were synapse (GO:0045202), postsynaptic membrane (GO:0045211), and synaptic membrane (GO:0097060); the top three significantly down-regulated MF enrichment terms were semaphorin receptor binding (GO:0030215), threonine-type peptidase activity (GO:0070003), and gated channel activity (GO:0022836) (Figure 1C).

In addition, compared to super-female *M. albus*, KEGG enrichment analysis of male *M. albus* showed (Figure 1D) 242 up-regulated pathways related to host genes associated with eccDNAs, with 9 pathways significantly up-regulated, including the calcium signaling pathway, SNARE interactions in vesicular transport, and phosphatidylinositol signaling system, etc. There were 235 down-regulated pathways related to host genes associated with eccDNAs, with 18 pathways significantly down-regulated, including progesterone-mediated oocyte maturation, purine metabolism, and basal transcription factors, etc.

### 3.3. Integrative Analysis of Host-Gene mRNA Expression and Differentially Abundant eccDNAs

**Figure 2:**
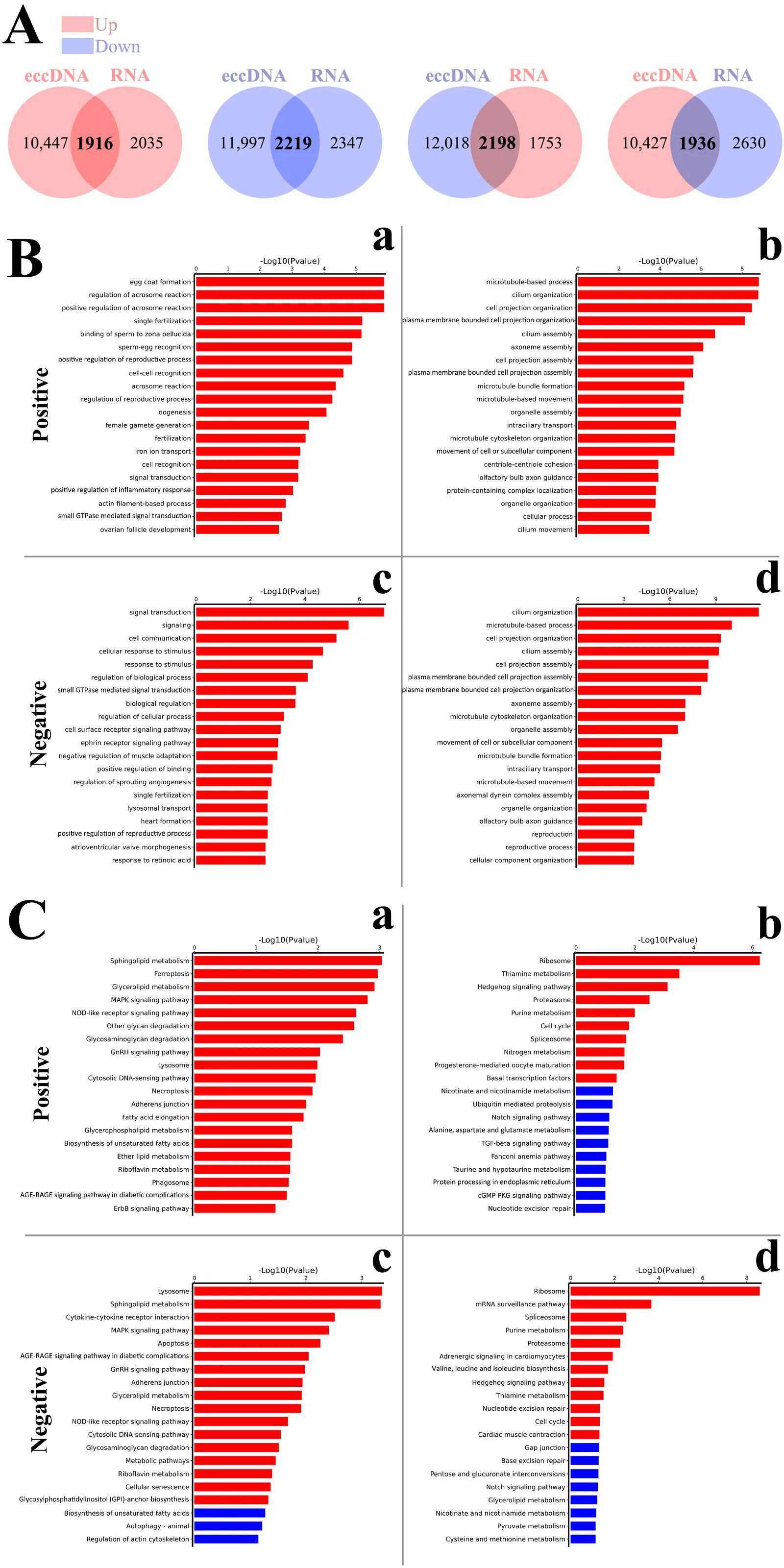
Integrative analysis of host-gene mRNA expression and differentially abundant eccDNAs. (**A**): Venn diagram visualization of overlapping gene sets between differential eccDNA abundance and mRNA expression. (**B**): GO biological process (BP) enrichment analysis: (**Ba**) genes with both increased eccDNA abundance and upregulated mRNA expression; (**Bb**) genes with both decreased eccDNA abundance and downregulated mRNA expression; (**Bc**) genes with decreased eccDNA abundance but upregulated mRNA expression; (**Bd**) genes with increased eccDNA abundance but downregulated mRNA expression. (**C**): KEGG pathway enrichment analysis: (**Ca**) genes with both increased eccDNA abundance and upregulated mRNA expression; (**Cb**) genes with both decreased eccDNA abundance and downregulated mRNA expression; (**Cc**) genes with decreased eccDNA abundance but upregulated mRNA expression; (**Cd**) genes with increased eccDNA abundance but downregulated mRNA expression. Red represents significantly enriched terms, and blue represents non-significant terms.

EccDNA sequencing data were integrated with previously published gonadal RNA-seq data of *M. albus*. The results of the Venn diagram (Figure 2A) revealed that there were 1916 overlapping genes showing up-regulation in both eccDNA abundance and mRNA expression, 2219 overlapping genes showing down-regulation in both eccDNA abundance and mRNA expression, 2198 overlapping genes in the group of eccDNA down-regulated and mRNA up-regulated, and 1936 overlapping genes in the group of eccDNA up-regulated and mRNA down-regulated.

Given that this study focuses on analyzing the biological activities in which genes are involved, particular attention was paid to the BP category of GO enrichment terms. The results of BP enrichment analysis (Figure 2B) are as follows: For the overlapping genes that were up-regulated in both eccDNA abundance and mRNA expression, the top three significantly up-regulated BP enrichment terms (Figure 2(Ba)) were vitelline envelope formation, regulation of acrosome reaction, and acrosome reaction. For the overlapping genes that were both down-regulated, the top three significantly down-regulated BP enrichment terms (Figure 2(Bb)) were microtubule-based processes, ciliary organization, and cell projection organization. In the group of eccDNA abundance down-regulated and mRNA expression up-regulated, the top three significantly enriched BP terms (Figure 2(Bc)) were signal transduction, signal transmission, and cell communication. For the overlapping genes where eccDNA abundance was up-regulated while mRNA expression was down-regulated, the top three significantly enriched BP terms (Figure 2(Bd)) were ciliary organization, microtubule-based processes, and cell projection organization.

KEGG enrichment analysis exhibited that in the case of genes up-regulated in both eccDNA abundance and mRNA expression (Figure 2(Ca)), there were 209 pathways of host genes associated with eccDNAs with 20 pathways significantly enriched, including sphingolipid metabolism, ferroptosis, and glycerolipid metabolism. For genes down-regulated in both eccDNA abundance and mRNA expression (Figure 2(Cb)), there were 161 pathways of host genes associated with eccDNAs, with 10 pathways significantly enriched, such as ribosome, thiamine metabolism, and the Hedgehog signaling pathway. In the group of eccDNA down-regulated and RNA expression up-regulated (Figure 2(Cc)), there were 212 pathways of host genes associated with eccDNAs with 17 pathways significantly enriched, including lysosome, sphingolipid metabolism, and cytokine-cytokine receptor interaction. In the group of eccDNA up-regulated while RNA expression was down-regulated (Figure 2(Cd)), there were 157 pathways of host genes associated with eccDNAs, with 12 pathways significantly enriched, such as ribosome, mRNA surveillance pathway, and spliceosome.

### 3.4. Validation of eccDNA

**Figure 3:**
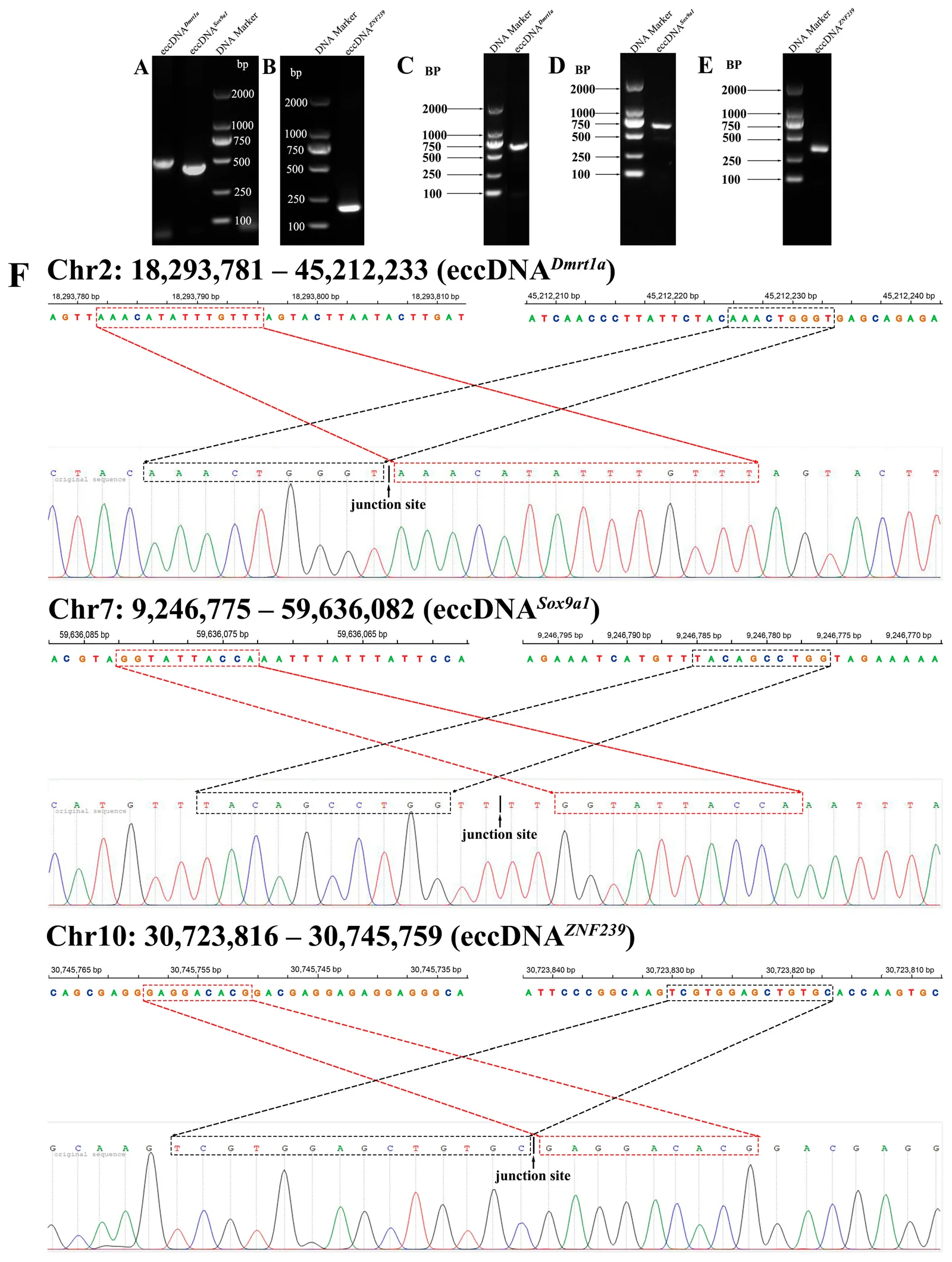
Validation of eccDNA. (**A**,**B**): electrophoresis images of PCR products from target gene sequences of eccDNAs; (**C**–**E**): electrophoresis verification images of PCR products from three gene-containing bacterial cultures; (**F**): Sequencing results of eccDNA junction sites. M: DNA Marker.

To validate the existence of eccDNA, we designed outward primers for eccDNA*^Dmrt1a^*, eccDNA*^Sox9a1^*, and eccDNA*^ZNF239^* (Table S1). Electrophoresis results (Figure 3A,B) of PCR amplification demonstrated successful amplification. Among the electrophoresis results (Figure 3C–E) of PCR amplification of single-cell bacterial lysates, we selected bacterial lysates with PCR products matching the size of the target sequences for sequencing. The sequencing results revealed that the flanking sequences at the junction sites of the three eccDNAs were consistent with the high-throughput sequencing results (Figure 3F), confirming the presence of these three types of eccDNA in *M. albus*.

### 3.5. mRNA-Level Expression and Transcript Localization of Dmrt1a, Sox9a1, and ZNF239 in the Developing Gonads of M. albus

**Figure 4:**
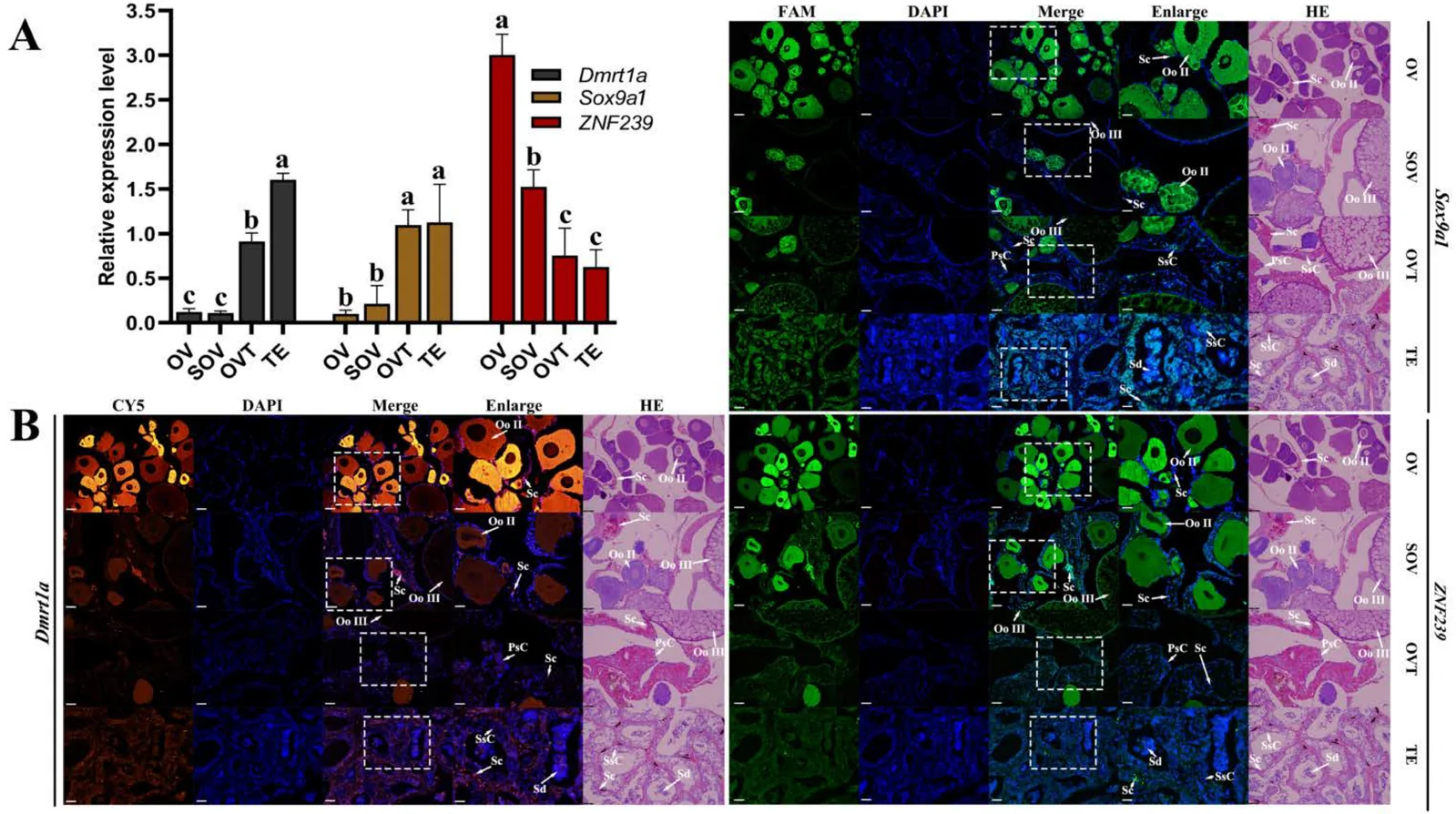
mRNA-level expression and transcript localization of target genes in developing gonads of *M. albus*. (**A**): Relative mRNA expression levels of *Dmrt1a*, *Sox9a1*, and *ZNF239* in developing gonads of *M. albus*. Different lowercase letters above bars indicate statistically significant differences between groups (*p* < 0.05). Groups sharing the same letter are not significantly different. (**B**): Cellular localization of *Dmrt1a*, *Sox9a1*, and *ZNF239* transcripts in gonadal cells. FAM (green signal)/CY5 (red signal) represents target gene transcripts; DAPI (blue signal) marks cell nuclei. White dashed boxes denote regions magnified in the Enlarge column. The scale bars for the FAM/CY5, DAPI, Merge, and HE columns are 50 μm, while the scale bar for the Enlarge column is 25 μm; Oo II, III: Oocytes at stage II, III; Sc: Somatic cell; Sg: Spermatogonia; PsC: Primary spermatocytes; SsC: Secondary spermatocytes; Sd: Spermatids; OV: Ovary; SOV: Super-female ovary; OVT: Ovotestis; TE: Testis.

RNA was extracted from the gonads of female, super-female, intersex, and male *M. albus* and then reverse-transcribed into cDNA. Primers were designed to amplify partial sequences of *Dmrt1a*, *Sox9a1*, and *ZNF239*. The results showed that the mRNA levels in males were significantly higher than those in females, and during the sex reversal process of *M. albus*, the expression levels of *Dmrt1a* and *Sox9a1* gradually increased. In contrast, *ZNF239* exhibited the opposite pattern, with a notably higher expression level in females than in males (Figure 4A). Similarly, FISH was performed on the gonads of female, super-female, intersex, and male *M. albus* for localization analysis. The results indicated that transcripts of *Dmrt1a*, *Sox9a1*, and *ZNF239* were detected in oocytes at stage II and III, granulosa cells, some somatic cells, and spermatocytes at various stages (Figure 4B).

### 3.6. Expression and Localization of Dmrt1a, Sox9a1, and ZNF239 in the Gonads of M. albus Subjected to AI Treatment

**Figure 5:**
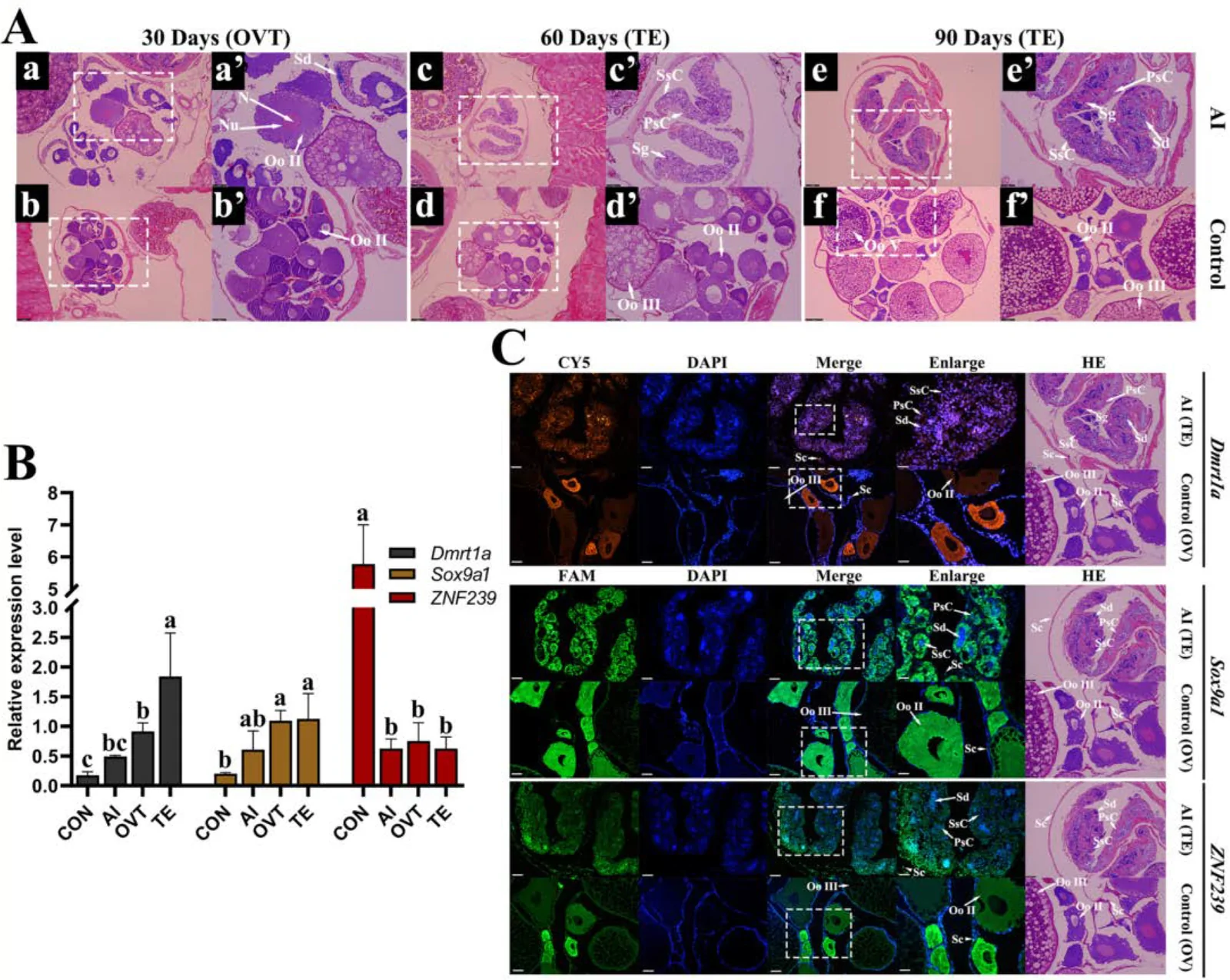
Histological changes, qRT-PCR results and transcript localization after AI treatment. (**A**): Gonadal histological transformations in *M. albus* following AI treatment; Sub-panels (**Aa**–**Af**) represent gonadal sections collected at sequential time points after AI treatment, and panels with prime symbols (e.g., (**Aa′**–**Af′**)) correspond to magnified regions marked by white dashed boxes in the parent panels. (**B**): Relative mRNA expression levels of target genes; Different lowercase letters above bars indicate statistically significant differences between groups (*p* < 0.05). Groups sharing the same letter are not significantly different. (**C**): Cellular localization of target-gene transcripts. FAM (green signal)/CY5 (red signal) represents target gene transcripts; DAPI (blue signal) marks cell nuclei. White dashed boxes denote regions magnified in the enlarged view. The scale bars for the original view columns are 50 μm, while the scale bar for the Enlarge column is 25 μm.

The AI-treated group and the control group of *M. albus* were sampled at 30 days, 60 days, and 90 days, respectively, and HE sections were prepared. The effects of sex reversal in juvenile *M. albus* after AI treatment are shown in Table 2. After 30 days of treatment in the AI-treated group, 2/5 of the female *M. albus* had their gonads transformed into intersex gonads. At this time, the number of stage II oocytes in the gonads decreased, some of the gonadal epithelial germ cells transformed into primary spermatocytes, and spermatids also appeared (Figure 5(Aa)). In contrast, after 30 days, the control group remained female, with a large number of stage II oocytes present in the ovaries (Figure 5(Ab)).

After 60 days of treatment in the AI-treated group, gonadal histology revealed that 2/5 of individuals exhibited intersex gonads and 3/5 developed male testes. At this time, the male *M. albus* had a large number of spermatogonia and primary spermatocytes, along with a small number of secondary spermatocytes (Figure 5(Ac)), indicating early-stage testes. After 60 days, the control group still remained female, with a large number of stage II oocytes and a small number of stage III oocytes present in the ovaries (Figure 5(Ad)).

After 90 days of treatment in the AI-treated group, all the *M. albus* had completely undergone sex reversal to males. At this time, the male *M. albus* had a large number of primary spermatocytes and secondary spermatocytes, with only a small number of spermatogonia and spermatids (Figure 5(Ae)), indicating that the testes were gradually maturing. After 90 days, the control group still remained female, with a large number of stage II and stage III oocytes, as well as a small number of stage V oocytes present in the ovaries (Figure 5(Af)), indicating that the ovaries were gradually maturing.

Gonads from the 90-day-treated group and the control group were collected for RNA extraction. After reverse-transcribing the RNA into cDNA, qRT-PCR was carried out for validation. The results showed that the expression levels of *Dmrt1a* and *Sox9a1* in the gonads of the AI-treated group were significantly higher than those in the control group but lower than those in naturally male *M. albus*. *ZNF239* showed the opposite pattern, with a significantly higher expression level in the control group than in the treated group (Figure 5B). The FISH results showed that transcripts of these three host genes were detected in stage II and stage III oocytes, granulosa cells, some somatic cells, and spermatocytes at various stages (Figure 5C).

## 4. Discussion

### 4.1. The Roles of Dmrt1a and Sox9a1 Genes in the Sex Reversal of M. albus

Numerous studies have demonstrated a close association between the *Sox9* gene in teleosts and testis development as well as male sex determination. Consequently, its expression level in male gonads is generally higher than that in female gonads. This viewpoint has been validated in various fish species, such as the gonochoristic *Astyanax altiparanae* [26] and the hermaphroditic *Lates calcarifer* [27] and *Epinephelus coioides* [28]. In *M. albus*, the expression level of *Sox9* continuously increases during the sex reversal process [29]. Similarly, *Dmrt1* also shows a high correlation with testis development and male sex determination. For instance, *Dmrt1* has been identified as the male sex-determining gene in *Cynoglossus semilaevis* [30] and *Collichthys lucidus* [31]. The expression patterns of *Dmrt1* in gonads can be mainly classified into two categories. One category is its specific expression in the testis, as observed in *Paralichthys olivaceus* [32] and *C. semilaevis*. The other category is its high expression in the testis with only trace amounts in the ovary, such as in gonochoristic *Acipenser baerii* [33] and *Acanthogobius ommaturus* [34], as well as in the hermaphroditic *Acanthopagrus schlegelii* [35].

Copy number variations have occurred in the genomes of many teleosts. In ricefield eels, *Sox9* is divided into *Sox9a1* and *Sox9a2*, which exhibit similar expression patterns, being expressed in the ovary, ovotestis, and testis [36]. On the other hand, due to alternative splicing, *Dmrt1* produces four isoforms, *Dmrt1a-d*. During the sex reversal process of *M. albus*, the expression levels of *Dmrt1a* and *Dmrt1b* in the gonads continuously increase, and the expression level of *Dmrt1a* is much higher than that of *Dmrt1b* [37]. Further research has shown that when primordial germ cells (PGCs) are knocked out in *M. albus*, testis-like structures form in their gonads. At 15 months of age, compared with the gonads of wild-type female *M. albus*, the expression levels of *Dmrt1* and *Sox9a* in the gonads of PGC-depleted *M. albus* significantly increase, which further indicates that *Dmrt1* and *Sox9a* are closely related to testis development in *M. albus* [8]. The results of this study also demonstrate that during the sex reversal process of *M. albus*, the expression levels of *Dmrt1a* and *Sox9a1* gradually increase, and their expression levels in males are much higher than those in females, which is consistent with previous research findings. In addition, FISH localization shows that *Dmrt1a* and *Sox9a1* do not have expression specificity in different types of gonadal tissue cells.

### 4.2. The High-Level Expression of ZNF239 in Female M. albus Is Related to Oocyte Maturation

*ZNF239* belongs to the zinc-finger protein family. However, the specific role of *ZNF239* in the regulation of gonadal development has not been reported so far. Previous studies have shown that *ZNF239*, as a DNA-binding repressor, can affect its transcriptional repression function when interacting with *laminA*/*C* and the nuclear matrix [38]. Many zinc-finger protein genes related to sex determination have been identified, such as *Mr-Masc* [39], *Bmznf-2* [40], *Zar1* [41], and *ZNF403* [42]. Among them, *Zar1* is highly expressed in the ovary and specifically expressed in stage I and stage II oocytes. The deletion of *Zar1* leads to the failure of the transition from oogonia to stage I oocytes and causes somatic cell apoptosis. In addition, homozygous mutants lacking *Zglp-1* exhibit female-to-male sex reversal, ultimately resulting in the development of fertile males [43]. The experimental results of this study indicate that the expression level of *ZNF239* in *M. albus* gradually decreases during the sex reversal process; that is, its expression level in females is much higher than that in males, suggesting that *ZNF239* promotes ovarian development. The expression level of *ZNF239* in juvenile *M. albus* is significantly higher than that in super-female *M. albus*, which may be due to the process of V-stage ovarian regression to III-stage ovarian regression in super-female *M. albus*. In conclusion, *ZNF239* is highly likely to promote ovarian development, and further in-depth research is still needed to elucidate its specific regulatory mechanism.

### 4.3. AI-Induced Effects on Gonadal Development and Expression of Sex-Related Genes in M. albus

Both 17α-methyltestosterone (MT) and aromatase inhibitors are widely used for masculinization in fish, but they induce masculinization through different mechanisms. MT, as an exogenous androgen, causes masculinization in fish by elevating androgen levels. Aromatase inhibitors, on the other hand, inhibit ovarian development and induce gonadal transformation towards testes by suppressing aromatase activity and thus reducing estrogen production [44]. Numerous studies have shown that high concentrations of MT or aromatase inhibitors can inhibit fish growth. Excessively high androgen levels following MT administration may lead to behavioral changes in male fish and inhibition of testicular development. Therefore, masculinization induced by aromatase inhibitors is generally considered more natural and effective [45,46]. Currently, letrozole, an aromatase inhibitor, has been experimentally validated in various hermaphroditic fish species, such as *Epinephelus marginatus* [47], *Plectropomus leopardus* [48], and *Amphiprion clarkii* with bidirectional sex-changing ability [49]. In this study, compared with the control group, *M. albus* treated with AI for 30 days showed a decrease in the number of stage II oocytes in the ovaries, with some gonadal epithelial germ cells transforming into primary spermatocytes and the appearance of spermatids, exhibiting typical intersex gonadal characteristics. After 60 days of treatment, oocytes in the gonads completely degenerated, forming cavities, while testicular structures such as seminiferous lobules and seminiferous tubules emerged. After 90 days of treatment, the gonads contained a large number of spermatocytes at various developmental stages, displaying typical testicular structures. In summary, the 2-month-old *M. albus* used in this study for AI treatment likely underwent sex reversal before their ovaries reached maturity, as female *M. albus* under natural conditions typically require 1–2 years to reach sexual maturity and spawn.

Regarding changes in the expression levels of sex-related genes in *M. albus* after AI treatment, previous studies have shown that, compared with the control group, estrogen levels in the blood of AI-treated *M. albus* significantly decreased, while testosterone levels markedly increased. Meanwhile, the expression levels of female-related genes were down-regulated, whereas the expression levels of male-related genes such as *Dmrt1* and *Sox9* were up-regulated [6]. Although this study did not measure blood indicators, compared with the control group, the expression levels of *Dmrt1a* and *Sox9a1* in the AI-treated group showed an upward trend. Previous studies have demonstrated that aromatase and estrogen play crucial roles in ovarian differentiation and development in various fish species. Therefore, a lack of aromatase and estrogen is likely to lead to sex reversal in fish [50]. Compared with naturally sex-reversed intersex gonads and testes, the expression levels of *Dmrt1a* and *Sox9a1* in the AI-treated group were lower, suggesting that the main cause of sex reversal in *M. albus* after AI treatment may be low estrogen levels rather than high expression levels of *Dmrt1a* and *Sox9a1*. Previous studies have indicated that *Dmrt1a* and *Sox9a1* are upstream regulatory genes in the second tier of gonadal differentiation, and in multi-level regulation, the inhibitory effect on the downstream aromatase gene has limited feedback on the expression of *Dmrt1a* and *Sox9a1*. Additionally, compared with the control group, the expression of *ZNF239* in the AI-treated group was significantly down-regulated, with no significant difference compared with naturally sex-reversed intersex gonads and testes, further suggesting that *ZNF239* may promote vitellogenesis and ovarian development.

### 4.4. eccDNAs Likely Play a Role in Sex Reversal of M. albus

Since their first discovery in 1965, eccDNAs have been detected in numerous animal and plant cells. These eccDNAs range in size from 100 to hundreds of thousands of base pairs, with the majority falling between 0.1–5 kb [21,51,52]. In this study, through whole-genome sequencing, an average of 62,574 and 34,562 eccDNAs were identified in super-female and male *M. albus*, respectively. These eccDNAs were mainly distributed in the range of 0.1–1 kb, peaking in the 250–500 bp interval. The distribution characteristics of eccDNAs in *M. albus* are generally consistent with those in other species, further supporting the view that small-molecular-weight eccDNAs are more easily formed.

Numerous studies have pointed out that eccDNAs carry partial or complete genes and possess the ability to encode functional proteins or transcribe RNAs to regulate organisms. For example, in the silk gland cells of silkworms, the eccDNA*^Fib-L^* was found to promote the transcription of the *Fib-L* gene [53]. A comparative analysis of eccDNAs in non-flying meat pigeons and flying carrier pigeons revealed that meat pigeons had approximately nine times more eccDNAs than carrier pigeons, and these eccDNAs were significantly enriched with genes related to energy metabolism, muscle formation, and maintenance in meat pigeons, such as *AGRIN* [20], resulting in meat pigeons having more muscle content. Compared with ordinary grass carp, the muscle tissues of slimming grass carp are enriched with a large number of eccDNAs directly related to muscle traits, making their meat more elastic [21]. In this study, by comparing the abundance levels of eccDNAs between super-female and male *M. albus*, 7881 down-regulated and 2738 up-regulated host genes associated with differentially abundant eccDNAs were found in male *M. albus*. Among them, through gene annotation corresponding to the genomic sequences of eccDNA loci and combined with previous studies on genes related to sex, three eccDNAs were selected for validation, and the existence of eccDNA*^Dmrt1a^*, eccDNA*^Sox9a1^*, and eccDNA*^ZNF239^* in *M. albus* was successfully confirmed. Although this study could not separate the mRNAs transcribed from these three eccDNAs from those transcribed from chromosomal DNA, based on the expression and localization changes of *Dmrt1a*, *Sox9a1*, and *ZNF239* during gonadal development in *M. albus*, combined with previous functional studies of eccDNAs in other species, it is inferred that these three eccDNAs may play a role in the sex reversal process of *M. albus*.

Furthermore, in this study, GO and KEGG enrichment analyses showed that these host genes associated with eccDNAs are closely related to regulatory functions such as biological regulation, regulation of cellular processes, regulation of biological processes, progesterone-mediated oocyte maturation, and basal transcription factors. In particular, the downregulation of the progesterone-mediated oocyte maturation pathway in male *M. albus* suggests that these eccDNAs play a role in inducing oocyte maturation, and their downregulation may lead to the transformation of *M. albus* gonads from ovaries to testes. Therefore, further exploration of the regulatory mechanisms of these three eccDNAs is needed in the future to more comprehensively reveal their specific functions in the sex reversal process.

## 5. Conclusions

(1) This study confirms that eccDNAs are also prevalent in *M. albus*, and eccDNA*^Dmrt1a^*, eccDNA*^Sox9a1^*, and eccDNA*^ZNF239^* are highly likely to play a role in the sex reversal process of *M. albus*.

(2) During the sex reversal process of *M. albus*, mRNA levels of *Dmrt1a* and *Sox9a1* were continuously up-regulated, and the expression levels in males were significantly higher than those in females, indicating that *Dmrt1a* and *Sox9a1* promote testicular development in *M. albus*. In contrast, mRNA abundance of *ZNF239* was significantly higher in females than in males, and its expression was extremely high in juvenile female *M. albus*, suggesting that it may promote vitellogenesis and ovarian development.

(3) Under 90 days of AI treatment, all female *M. albus* were transformed into males. During this process, mRNA levels of *Dmrt1a* and *Sox9a1* were up-regulated, while mRNA levels of *ZNF239* were down-regulated. Compared with naturally sex-reversed ovotestes and testes, the expression levels of *Dmrt1a* and *Sox9a1* in the testicular tissues of the AI-treated group were lower, indicating that the sex reversal in *M. albus* induced by AI treatment is mainly due to the inhibition of aromatase gene activity, which is located downstream in the sex differentiation system, leading to a decrease in estrogen levels. The inhibition of aromatase activity has limited feedback on the upregulation of the expression levels of the upstream *Dmrt1a* and *Sox9a1* genes.

In summary, the results of this study indicate the presence of eccDNA*^Dmrt1a^*, eccDNA*^Sox9a1^*, and eccDNA*^ZNF239^* in *M. albus*, and they are highly likely to play a role in the sex reversal process of *M. albus*. In addition, this study is the first to reveal the potential role of *ZNF239* in ovarian tissue.

## Figures and Tables

**Table 1:** Statistics of Raw Reads, Clean Reads, and Q30.

Sample Name	Raw Reads	Clean Reads	Q30 (%)
SupOV1	138,371,756	134,541,126	92.94
SupOV2	139,095,932	136,161,978	93.20
SupOV3	134,531,860	131,553,790	93.91
TE1	138,643,378	136,049,258	93.56
TE2	144,042,580	139,981,824	93.32
TE3	148,533,720	145,706,884	93.11

**Table 2:** The effect of sex reversal *M. albus* after AI (500 mg/kg) treatment.

Day of AI Treatment	Total Sample (Number)	Male Fish		Intersex Fish		Female Fish	
		Sample (Number)	Proportion (%)	Sample (Number)	Proportion (%)	Sample (Number)	Proportion (%)
30 days	5	0	0%	2	40%	3	60%
60 days	5	2	40%	3	60%	0	0%
90 days	3	3	100%	0	0%	0	0%

## Data Availability

The previously generated gonadal RNA-seq raw data used for integrative analysis are available in the NCBI SRA under BioProject accession PRJNA1292716. Sequence alignment was performed using the *Monopterus albus* reference genome assembly GCF_001952655.1 (M_albus_1.0) downloaded from NCBI RefSeq. All other relevant datasets supporting the findings of this study are included within the article and its Supplementary Materials.

## Supplementary Materials

The following supporting information can be downloaded at: https://www.mdpi.com/article/10.3390/biom16101480/s1, Supplementary Figure S1: Schematic overview of the wet-lab experimental procedures and bioinformatic workflow for eccDNA identification and differential abundance analysis in *M. albus*; Figure S2: Bubble Plot for GO Enrichment Analysis of host genes associated with differentially abundant eccDNAs; Figure S3: Bubble Plot for Pathway Enrichment Analysis of host genes associated with differentially abundant eccDNAs; Table S1: Primers used in this study.
