# Supplementary material for: Role of Extrachromosomal Circular DNA in Sex Reversal of Monopterus albus
Source: Biomolecules. 2026 Oct 12;16(10):1480. doi: 10.3390/biom16101480 (PMC13702476; doi:10.3390/biom16101480)
Supplement: Supplementary file 1 [file biomolecules-16-01480-s001.zip › biomolecules-4505186-supplementary.pdf]

Role of Extrachromosomal Circular DNA in Sex Reversal of *Monopterus albus*

Junchao Fang [1,2], Guanglve Li [1], Wenyin Luo [1], Haifeng Tian [2] and Qiaomu Hu [1,2,*]

[1] State Key Laboratory of Mariculture Biobreeding and Sustainable Goods, Yellow Sea Fisheries Research Institute, Chinese Academy of Fishery Sciences, Qingdao 266071, China; 15119438380@163.com (J.F.); 13659644863@163.com (G.L.); ioop4916@163.com (W.L.)

[2] Yangtze River Fisheries Research Institute, Chinese Academy of Fishery Sciences, Wuhan 430223, China; tianhf@yfi.ac.cn

* Correspondence: hqmu0806@163.com

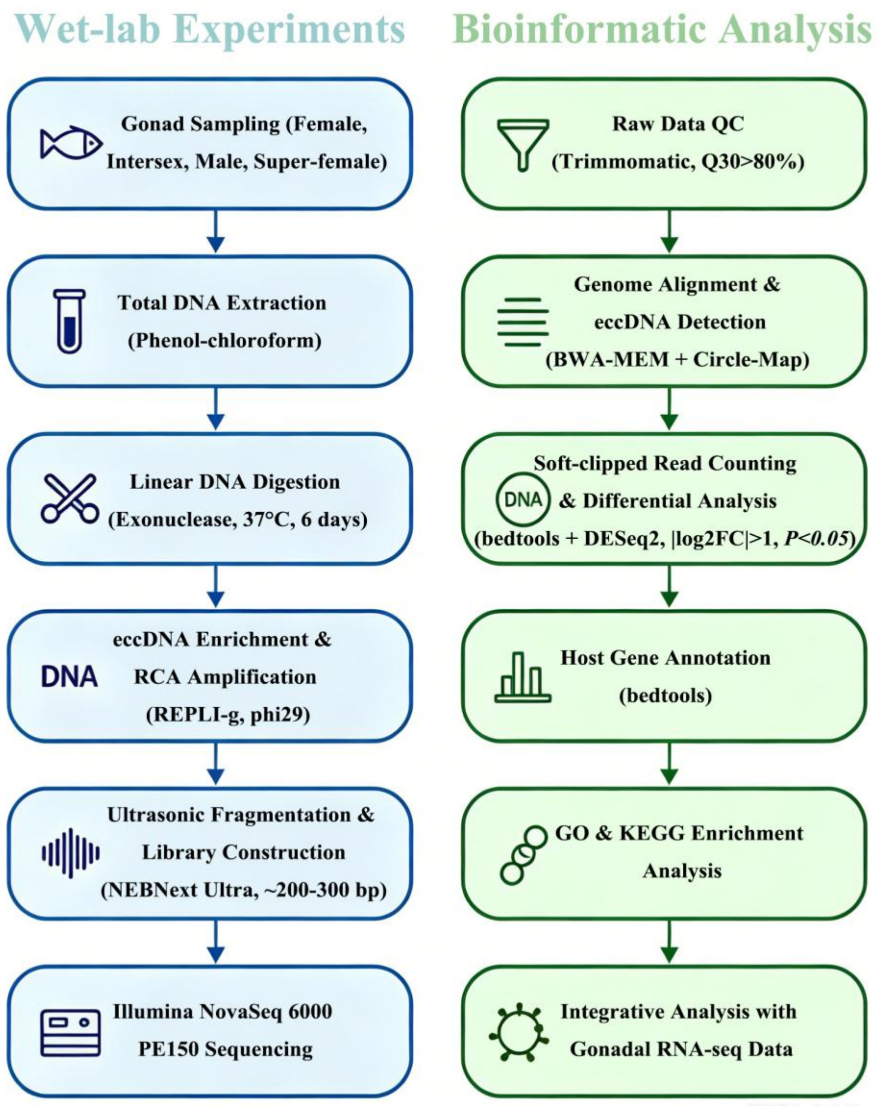

Figure S1: Schematic overview of the wet-lab experimental procedures and bioinformatic workflow for eccDNA identification and differential abundance analysis in *M. albus*.

The left panel shows the wet-lab workflow: gonad sampling, total DNA extraction, linear DNA digestion, eccDNA enrichment and RCA amplification, ultrasonic fragmentation, library construction, and Illumina NovaSeq 6000 PE150 sequencing. The right panel shows the bioinformatic pipeline: raw data quality control, genome alignment and eccDNA detection, soft-clipped read counting and differential abundance analysis, host gene annotation, GO/KEGG enrichment, and integrative analysis with published gonadal RNA-seq data.

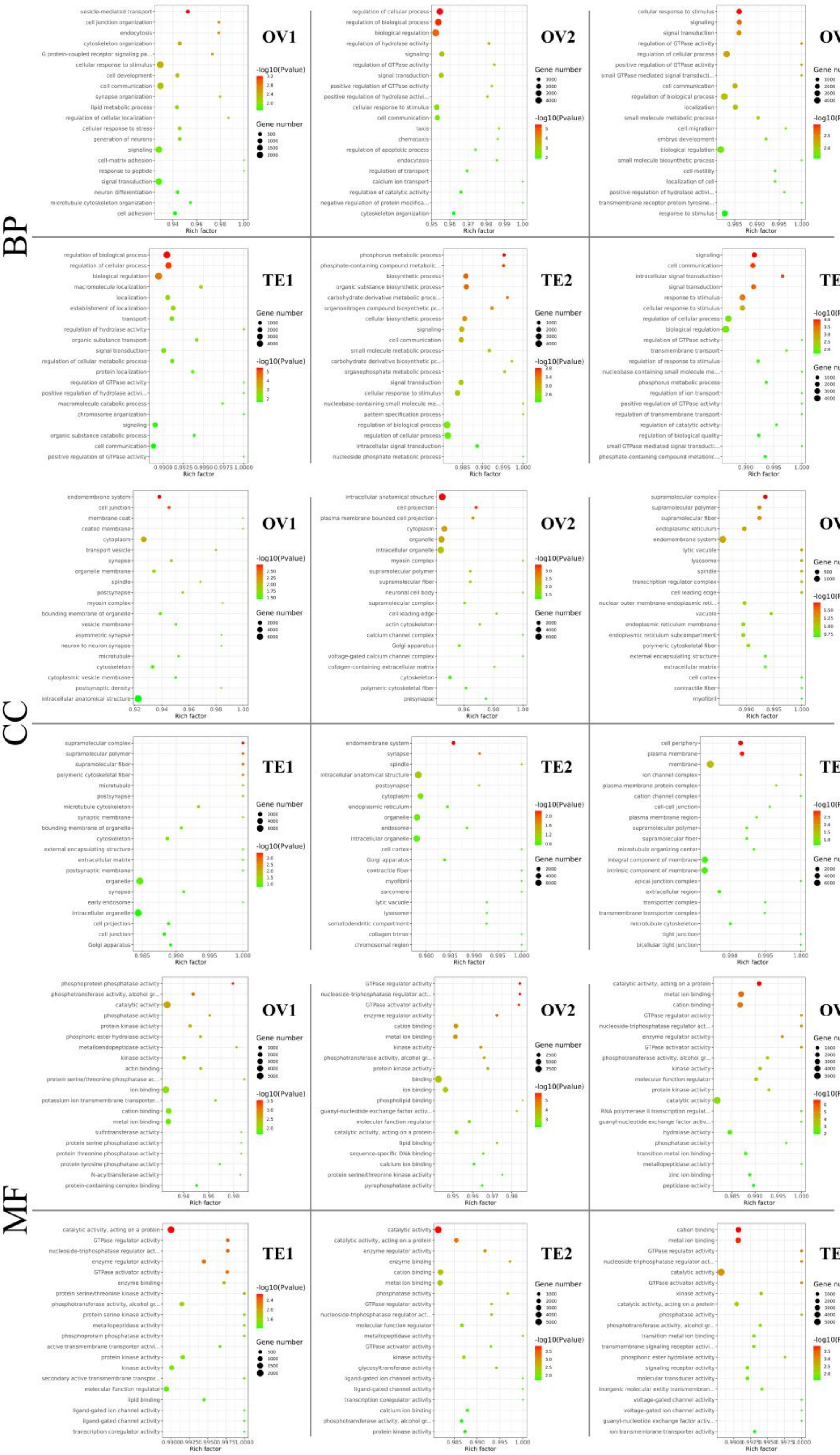
BP
CC
MF
OV1
OV2
OV3
TE1
TE2
TE3
Rich factor
-log10(Pvalue)
Gene number

Figure S2: Bubble Plot for GO Enrichment Analysis of host genes associated with differentially abundant eccDNAs

The y-axis represents GO terms; the x-axis represents the Rich factor. The size of the dots indicates the number of host genes associated with differentially abundant eccDNAs enriched in each GO term, while the color denotes the significance P-value of the GO term, with smaller P-values indicating stronger enrichment significance. BP: Biological Process; CC: Cellular Component; MF: Molecular Function; SOV 1-3: Ovarian samples 1-3 from Super-female individuals; TE 1-3: Testis samples 1-3.

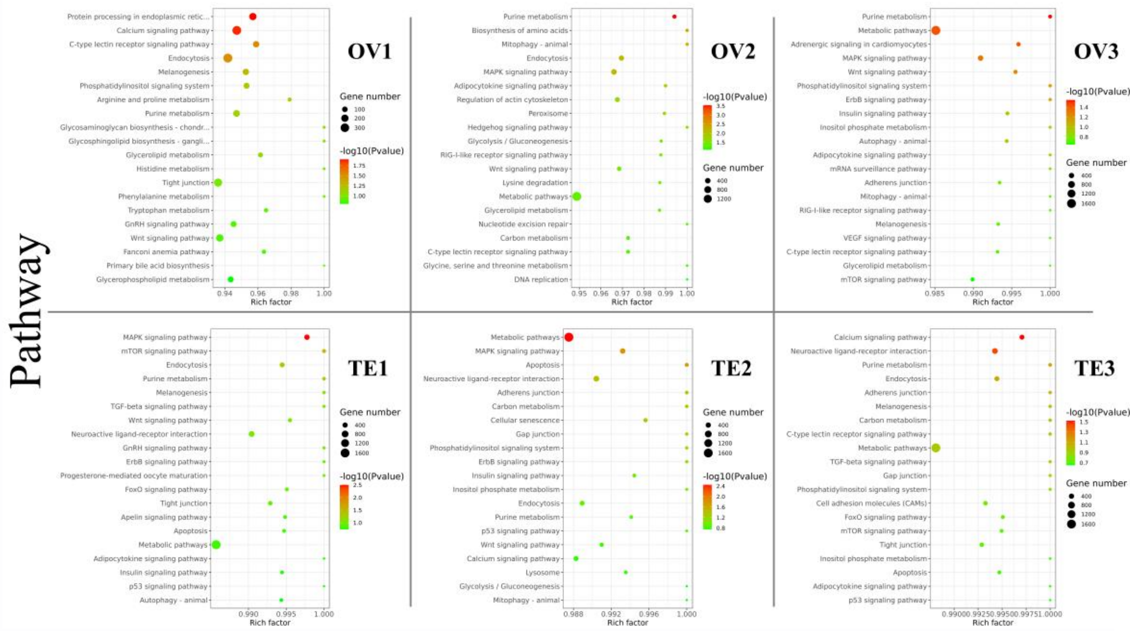

Figure S3: Bubble Plot for Pathway Enrichment Analysis of host genes associated with differentially abundant eccDNAs

The y-axis represents Pathway terms; the x-axis represents the Rich factor. The size of the dots indicates the number of host genes associated with differentially abundant eccDNAs enriched in each Pathway term, while the color denotes the significance P-value of the Pathway term, with smaller P-values indicating stronger enrichment significance. SOV 1-3: Ovarian samples 1-3 from Super-female individuals; TE 1-3: Testis samples 1-3.

Table S1 Primers used in this study.

Gene name	Forward primers (5’−3’)	Reverse primers (5’−3’)	Amplified length (bp)	Purpose
eccDNA^{Dmrt1a}	GCAGAATTATCAGTA<br>AACACACGGT	AGGGCTCTTCACTTT<br>CTTGCTG	492	PCR
eccDNA^{Sox9a1}	AGCCCACAGAAATGC<br>TTGTAAAT	GCAATCACTTTTTCA<br>TTTCACTTG	443	PCR
eccDNA^{ZNF239}	CCACTGTTCGGCTCA<br>CGG	TGGTCCAGCCGCAG<br>GAAT	180	PCR
MA*Dmrt1a*	CTGCCAGTGCTCCAA<br>ATGTAAA	GTAGGCGTCGGGTT<br>TCGTC	201	qRT-PCR
MA*Sox9a1*	CCTCCTCTTACCCCTC<br>CATCA	GTCGGCGATTGGGG<br>TGTA	164	qRT-PCR
MA*ZNF239*	GAAGCTGAAGAGGC<br>AGAAGAAAA	ACAGAGGTGAAGCT<br>TTATTCTGGTT	139	qRT-PCR
MAEF-1a	CGCTGCTGTTTCCTTC<br>GTCC	TTGCGTTCAATCTTC<br>CATCCC	102	qRT-PCR
MA*Dmrt1a*	CTCTCGGCTATCAGTTTACATTTG<br>CATCTCTGATACACACGGG		24	Fish
MA*Sox9a1*	GAGGTTTGTTTTTAGTTGAGCCGTTGA<br>CTTCCACAAACGGACGCTTTTCTACT		94	Fish
MA*ZNF239*	ACTGTGCTCTTGTGATGGAGGG<br>AAGACCGTGATGTTGAGAAGGCCTTGTCACA<br>GTGCTGACAGAGGTGAAGC		50	Fish
